# Do schools differ in suicide risk? the influence of school and neighbourhood on attempted suicide, suicidal ideation and self-harm among secondary school pupils

**DOI:** 10.1186/1471-2458-11-874

**Published:** 2011-11-17

**Authors:** Robert Young, Helen Sweeting, Anne Ellaway

**Affiliations:** 1 MRC Social and Public Health Sciences Unit, University of Glasgow, 4 Lilybank Gardens, Glasgow G12 8RZ, UK

## Abstract

**Background:**

Rates of suicide and poor mental health are high in environments (neighbourhoods and institutions) where individuals have only weak social ties, feel socially disconnected and experience anomie - a mismatch between individual and community norms and values. Young people spend much of their time within the school environment, but the influence of school context (school connectedness, ethos and contextual factors such as school size or denomination) on suicide-risk is understudied. Our aim is to explore if school context is associated with rates of attempted suicide and suicide-risk at age 15 and self-harm at age 19, adjusting for confounders.

**Methods:**

A longitudinal school-based survey of 1698 young people surveyed when aged 11, (primary school), 15 (secondary school) and in early adulthood (age 19). Participants provided data about attempted suicide and suicide-risk at age 15 and deliberate self-harm at 19. In addition, data were collected about mental health at age 11, social background (gender, religion, etc.), and at age 15, perception of local area (e.g. neighbourhood cohesion, safety/civility and facilities), school connectedness (school engagement, involvement, etc.) and school context (size, denomination, etc.). A dummy variable was created indicating a religious 'mismatch', where pupils held a different faith from their school denomination. Data were analysed using multilevel logistic regression.

**Results:**

After adjustment for confounders, pupils attempted suicide, suicide-risk and self-harm were all more likely among pupils with low school engagement (15-18% increase in odds for each SD change in engagement). While holding Catholic religious beliefs was *protective*, attending a Catholic school was a *risk *factor for suicidal behaviours. This pattern was explained by religious 'mismatch': pupils of a different religion from their school were approximately 2-4 times more likely to attempt suicide, be a suicide-risk or self-harm.

**Conclusions:**

With several caveats, we found support for the importance of school context for suicidality and self-harm. School policies promoting school connectedness are uncontroversial. Devising a policy to reduce risks to pupils holding a different faith from that of their school may be more problematic.

## Background

An act of suicide is influenced by both individual risk (e.g. pre-existing mental health problems or stress) and the social and environmental context in which an individual is embedded (e.g. a supportive environment and prevailing moral norms against suicide) [1]. During the 'teen years' the incidence of suicide attempts, suicide ideation and self-harm peak [2] and suicide is a leading cause of death [1]. This is probably attributable to the increased individual risk factors associated with this transitional life-stage, such as pubertal changes, increased stress, depression and impulsiveness, although background factors such as family situation or socioeconomic circumstances are all important predictors [1]. During this period there are two major sources of environmental or contextual influence; school and neighbourhood. Given the amount of time young people spend in each [3], even a small effect may have a pervasive influence, since contextual factors may influence the behaviour of an entire community, rather than a single individual.

In this paper we explore the association between the school (school connectedness, denomination, school ethos, size of school) and suicide-risk, adjusting for important background factors, such as perception of local neighbourhood and prior mental health risk. Our data are derived from a longitudinal study of approximately 1700 young people followed from age 11 to 19, located within 43 different schools and their associated neighbourhoods.

We frame our analysis using three theories relevant to contextual influences on suicidal behaviour; the Ecological-transactional model; Durkheim's anomic-egoistic theory and Joiners transpersonal theory of suicide. However, before outlining each theory, we briefly review the relevant literature on major contextual influences on suicide, how these are conceived, measured and the potential difficulties inherent in contextual analysis.

### Neighbourhood, contextual effects and suicide

Contextual influences on suicide rates are well known: rates of suicide vary by access to firearms and geographical location, [4] and concepts such as *anomie *(the disconnect between individual and societal norms and expectations) are classic contextual explanations of suicide [5]. A number of studies have investigated the phenomenon of geographical clustering in young adult [6] and teenage suicides [7]. Although the mechanisms involved in the epidemiology of suicide clusters are still unclear, if one excludes the possibility of contagion, explanations of contextual effects can be putatively linked to standard risk factors such as social inequalities, inequalities in social capital or 'social connectedness', cultural or religious differences and statistical artefact due to compositional effects [1]. In relation to self harm, some studies have found rates of hospitalisation due to self harm to be higher in deprived areas [8-10], but a large part of contextual effects seem attributable to compositional factors.

### Neighbourhood and school influences on mental health

The neighbourhood is associated with a variety of child and adolescent health outcomes. A systematic review of multi-level studies estimated that, after accounting for individual and family characteristics, neighbourhood determinants explain approximately 10% of the variance in child and adolescent health outcomes [11]. How young people themselves perceive and experience their local physical and social neighbourhood (e.g. the degree of attractiveness or level of amenities) is related to a range of mental health outcomes [12,13].

Studies which explore the contextual influence of school on suicidal behaviour and self-harm are rare, although we can extrapolate to a degree from school studies of substance use [14] and wellbeing [15]. When reviewing the influence of school from a multilevel context including four studies of 'problem behaviour and wellbeing' outcomes, the intra class correlations (ICC; the percent of variance attributable to contextual influence) never exceeded 8% and most were below 4% [15]. Therefore, it is likely that the contribution of school in relation to suicidal behaviour is small, but nonetheless important given the severity of the outcome and the implications for policy development.

### School connectedness, school ethos and school context

Positive orientation to school, teacher support, school engagement, school attachment, school bonding, school climate, school involvement, and school connectedness are all terms that refer to the attachment individual pupils have to the school and which have been measured with a variety of scales [16]. Although there are differences in focus, most of these questionnaires arguably tap a similar underlying construct we term *school connectedness*. School connectedness is associated with many health behaviours [17], among them measures of psychological distress and suicidality. For example, a recent study of school factors among Norwegian adolescents found teacher support strongly predicted lower depressive symptoms both cross-sectionally and longitudinally at one-year follow-up [18].

Compared to school connectedness, the concept of *school ethos *is more nebulous, described by Hughes [19] as "... a convenient word to use about a school, as long as one doesn't define it." It generally refers to the overall school culture, atmosphere or climate - arguably equally vague terms. Despite measurement problems, many researchers consider school ethos important, with ethos operationalised as the sum effect of school processes and relationships [3]. A composite measure of school ethos was constructed for previous analyses of the dataset used in this paper using multiple indicators [14]. Factor analysis of a range of pupil-reported school-based items produced four measures: school environment (e.g. quality of the physical environment); pupil involvement (e.g. feeling part of the school); pupil (dis)engagement (e.g. dislike of school); and quality of teacher-pupil relationships (e.g. number of 'good' teacher/pupil relations). Measured at the individual level, these can be considered forms of school connectedness or engagement, when measured at the school level they are more accurately described as indicators of school ethos, and accordingly, these four variables were aggregated across all pupils in each school to produce an overall contextual 'school ethos' measure. Each measure significantly correlated with at least one form of substance use and, given the associations between school connectedness and psychological health, it is plausible these measures of school environment may be associated with suicide attempts and self-harm.

*School context *can refer to both aggregate measures of individual perceptions of school connectedness (as described above) and more 'objective' characteristics of the school - something that is a true property of the school such as its size, roll, or denomination. In both educational and health research, school size is a factor related to school involvement, alienation and isolation [3]; smaller schools tend to be associated with smaller communities and may provide a more 'connected' environment with greater opportunity to 'know' both teachers and fellow pupils [20]. A counterargument is that, while larger schools may be less cohesive they offer greater educational variety and the opportunity for pupils to develop more specialised and supportive ciques with similar interests.

Within the West of Scotland context, the link between individual religion and school is an atypical one, because in some regions Scottish schools are partially stratified according to their pupil's religious background. In general, pupils from a Catholic background attend what are termed denominational schools, although this is not prescriptive and a minority of pupils from non-catholic backgrounds do attend denominational schools. Non-denominational schools on the other hand make no distinction on religious grounds and accept pupils from any religious background, although, within the West of Scotland the majority of pupils come from a nominally Protestant or 'Church of Scotland' background. In general, pupil religion and school religious denomination *match*, thus pupils with Catholic parents attend Catholic schools, while pupils with protestant parents attend non-denominational schools. However, within the West of Scotland this does not happen in every case and a minority (3-4%, denominationally *mismatched *pupils) do not conform to this pattern, Thus, due to a range of circumstances, a minority of pupils who would normally attend a non-denominational school, attend a Catholic school, and a minority of Catholic pupils attend a non-denominational school. In respect of school denomination, individual religion or religiosity is (generally) considered to lower the risk of, those without a religious preferences or minority religions developing mental health problems, but whether or how school and individual religion interact is unknown [21-23].

### Religion, mental health and suicide risk

Despite secular trends, religion and religious institutions remain an enduring component of individuals' lives and both are linked to mental health in a complex fashion [17,18]. Although the evidence is not indisputable, the overall conclusion is that religious belief or religiosity is 'good' for mental health and reduces suicide-risk, although the exact mechanisms are unclear [22]. Among the likely explanations are moral prohibition (most religions explicitly prohibit or disapprove of suicide), improved coping mechanisms (a hopeful or positive orientation), increased social support (associated with religious institutions and communities), increased sense of community (through shared values and orientation) and increased pastoral care [21-23].

The link between regular church attendance and mental health at age 11 was explored in analyses based on the dataset which forms the basis of this paper [23]. This demonstrated that church attendance interacted with religious denomination, such that weekly (compared with less frequent) church attendance was associated with a mental health advantage (increased self-esteem and reduced anxiety or depression) for Catholics or disadvantage for children with a Church of Scotland affiliation. To date, the bulk of research on mental health and religion has been focused at the individual level and an important remaining task is to differentiate between the individual and contextual effects of religion. In other words, we need to contrast the effects of a religious belief with the effects of living within a religious environment. The two influences are usually confounded, but our school study can partially disentangle these components. Psychological and sociological theories stress the importance of such contextual factors and their likely mechanism of influence.

### The ecological-transactional framework

One major psychological theory that incorporates contextual influence is the ecological-transactional model (E-TM). The E-TM has been used to explain a range of behaviours affected by ecological context such as child maltreatment [24], community violence [25], sexual behaviour [26] and most relevant to the current study, suicidal behaviours [27]. Briefly, E-TM explains behaviour on four distinct hierarchically ordered, but interacting, 'ecological levels'; specifically macrosystem; exosystem; microsystem and ontogenic levels. The macrosystem focuses on overarching or societal level factors such as general values and cultural beliefs. The exosystem emphasises the influence of intermediate, but still large social groupings such as neighbourhoods and communities, typically measuring factors such as community norms and exposure to risk at the neighbourhood level. The microsystem concentrates on the influence of smaller social groups and communities, which can vary dramatically in size, but includes structures such as school, peer group and, at the finest level, family groups. Examples of risk factors at this level are school/workplace ethos, peer-group norms and exposure to risk at the family level. Finally, the ontological level represents individual variation and includes typical psychosocial variables such as coping style or capacity for emotional regulation. It should be apparent that while a useful conceptual model, incorporating every ecological level into a single analysis is unfeasible. Nevertheless, the E-TM's greatest contribution is to emphasise the importance of contextual factors and consideration of the most appropriate 'level of influence' relevant to each outcome and lifecourse stage.

As a theory the E-TM is open to criticism and can be considered more a 'conceptual framework' than a general theory, because without expert interpretation it can make few specific predictions. For example, in relation to reducing suicide among Native Americans, researchers using the E-TM suggest that broad-based community-level interventions might be more effective, because the roots of psychopathology are at the ecological rather than individual-level [28]. They propose that Native American teenagers' identity - positioned betwixt native and modern cultures - leads to a disrupted sense of social connection and greater risk of suicide, and that strengthening family ties, cultural bonds and native cultural practices may reduce such risk. A recent longitudinal study used the E-TM to examine the influence of the microsystem (parental, peer and school) on attempted suicide among American adolescents [27], with a particular focus on the interaction between individual and protective contextual effects such as family, peer and school 'connectedness'. Somewhat counter to expectation, it found no main effects for school and peer connectedness and only one, rather complicated interaction, interpreted as suggesting that boys who had previously attempted suicide, with poor peer relations, but good school connectedness were at lower than expected risk of further suicide attempts. Thus, the E-TM would predict a protective main effect for parental support, but few significant interactions.

### A multilevel approach to Durkheim's theory of suicide

The E-TM is a very general framework, but key theories specifically designed to explain suicide explicitly recognise the importance of social context. Emile Durkheim proposed an influential and comprehensive contextual theory of suicide. Briefly, Durkheim's theory proposes two forms of suicide, individually and socially motivated [5]. Durkheim suggested that conventional psychiatric research overestimates individual factors (mental illness and other psychologically orientated explanations) and underestimates societal and contextual influences on suicidality. He focused on the relationship which societal regulation and social integration had with suicide and outlined several types of suicide, but the two most relevant and recognised are *egoistic*, and *anomic*.

Egoistic suicide arises when an individual's connection to society is fragile, or where circumstances, such as the death of a partner or family break-up, weaken these bonds. Thus, family stability is highly important, but other sources of social connection such as school connectedness may offer a buffer against egoistic suicide. Anomic suicide is bound to the concept of 'anomie' - a disturbing mismatch between individual and societal norms, values, expectations and aspirations. Durkheim argues that during times of social flux, anomie increases, leading to higher rates of suicide. The degree of anomie will vary between different societies or institutions depending on the ethos promoted; those with a high degree of consensus, connection and integration between individual and social norms and institutions should have lower risks of suicidal behaviours. Schools are one type of institution where the degree of regulation and control, school ethos and school connectedness varies systematically. Traditional religion may also provide the shared values and norms that reduce risk of anomic suicide. Since suicide, attempted suicide and self-harm are predicted by many of the same biopsychosocial factors, focusing on more common forms of 'suicidality' should provide similar (although qualified) insights into the links between context and suicide risk as studies which focus on completed suicide [29].

At least one paper has taken a multi-level approach to youth and suicidality in relation to Durkheim's theory of suicide [29]. That American study looked at levels of social integration at the neighbourhood level and adjusted for individual background, such as parental supervision and school attachment, with a particular focus on the protective effects of religiosity. An important finding concerns the cross-level interaction between neighbourhood measures of religiosity and depression on attempted suicide, which indicated that depressed adolescents in secular neighbourhoods are at greater risk of suicide, compared to those in neighbourhoods that are religious. The authors attribute this protective effect to the greater availability of social and emotional support, stronger social bonds and the additional coping mechanisms available in such neighbourhoods that act as a buffer against suicide and depression during 'turbulent adolescence' when identity and values are formed.

While that study is exemplar in its methodological rigour, it is focused on the exosystem level (neighbourhood) and limited to a single outcome - attempted suicide. We extend this approach, using a similar design, but focusing on the microsystem level (school) and incorporating additional measures of suicide risk such as deliberate self-harm and suicidal thoughts or plans. We also have the opportunity to explore the interaction between individual religion and school denomination.

### Joiners Interpersonal Theory of Suicide

Thomas Joiners Interpersonal Theory of Suicide (ITS) is arguably, the most comprehensive modern theory of suicide available and draws heavily on Durkheim's concepts of anomie and alienation. The theory proposes that a lethal or near lethal suicide attempt requires the presence of three factors; *thwarted belongingness *(perceived loneliness or exclusion); *perceived burdensomeness *(perceived low self- or social-worth) and *capability for suicide *(past experience of or exposure to self-injury and suicide). The first factor, thwarted belongingness, is of primary relevance to this study and is broadly compatible with Durkheim's concept of anomie. Thus, the present study fits well with both classic sociological and current psychological theories of suicide which emphases perceived social connection - the inverse of thwarted belongingness.

### Aims

Using a large representative longitudinal sample of young people, surveyed in primary school and both during and after secondary school, we address three aims. First, to confirm if the significant associations between attempted suicide, suicide risk or self-harm and either school connectedness or school-level variables, found in prior studies, remain after adjustment for relevant individual level confounds; second, to explore the possible cross-level interaction between individual religion and school denomination suggested by both ET-M and Durkheim's anomies theory; and third, to estimate what proportion of attempted suicide, suicide risk and self-harm can be attributed to the secondary school, an important ecological level within the ET-M and other theories relevant to suicidality.

## Methods

The material for the study is drawn from the 'West of Scotland 11 to 16/16+ Study' [30], a longitudinal community health and lifestyle survey of young people, administered first in schools via questionnaire and then after leaving school by nurse interview. The focus here is on data collected in 1994-5, (age 11) 1999 (aged 15) and 2002-4 (age 19). The study received approval from Glasgow University's Ethics Committee, participating Education Authorities and schools. Informed consent was obtained from the parents of all participants via 'opt-out' consent forms at ages 11 and 15, oral consent from participants at each wave and written consent at age 19.

Due to the school-based nature of the study, the sampling scheme involved several elements to ensure a representative sample at both the primary and secondary school stages and sufficient school units to explore school effects using multilevel modelling methods [31]. Briefly, the survey used a reverse sampling procedure which randomly selected 43 secondary schools stratified by religious denomination and deprivation, with a separate stratum for independent vs. local authority run schools. These 43 secondary schools were used to select a random sample of 135 primary schools, comprising 'feeder schools', together with those making a high number of placing requests. In contrast to some European or North American school systems, 'feeder primary schools' do not usually share a campus with a target secondary school, thus pupils from every primary school may be equally prone to experience some degree of school transition problems [32]. From these primary schools, classes were randomly selected with all pupils in the classes eligible to participate. Full details of the sampling strategy are available elsewhere [31]. Of 2793 target, pupils attending the 43 secondary schools, 2586 (93%) participated in the baseline (age 11) survey. At age 15, the number of participants reduced to 2196 (79%), with losses in the post-school period reducing the sample size at age 19 to 1258 (45%). At age 15 1,860 (67%) respondents completed selected modules of a self-administered computerised (Voice) version of the Diagnostic Interview Schedule for Children (DISC) [33] which included a section on suicidal thoughts and behaviours [34]. A section about current and previous self-harm was included in the nurse administered interview at age 19.

At age 11, the sample was representative (in terms of sex and social class composition) of 11 year olds in the study area [35]. Differential attrition made later waves less representative, with attrition greater among lower social class groups, school truants, pupils of lower ability and with greater emotional and behavioural problems. To compensate for these biases, a weighting scheme was derived [35]. Use of these weights did not substantively alter the results and unweighted results are presented. The data used in this paper refer to 1,860 pupils who completed the psychiatric component and in their final year of compulsory education in 43 mainstream secondary schools in the Glasgow area, 1256 of whom provided information when aged 19. Almost all parents provided information on pupils' religious background and family socioeconomic status via a supplementary questionnaire in the baseline (age 11) of the study. After excluding those with missing data in other variables, 1698 cases were available for analysis of suicide risk at age 15, and 982 for self-harm at age 19. Due to low numbers, one school was omitted from the analysis.

### Measures

#### Suicidality: attempted suicide, Suicide risk and self-harm

As part of the 1999 (age 15) psychiatric interview, pupils were asked, 'In the last year, was there a time when you thought seriously about killing yourself?' and 'Have you ever, in your whole life, tried to kill yourself or make a suicide attempt?' A 'yes' response to either question was categorized as 'suicide risk' at age 15 and any report of an attempt to 'kill yourself' categorized as a 'suicide attempt'(ever).

The 'nadir' or highest risk model of suicide prediction has considerable empirical support [36]. Its main proposal is that the likelihood of an imminent suicide attempt is (to some degree) contingent on both past and recent suicidal thoughts and behaviors, and this risk increases as cognitions become more recent, concrete and goal directed, with prior experience of attempting suicide the highest risk. An age 15 'suicide risk' score, range 0 (least risk) to 5 (most risk), was created using 5 binary items from the psychiatric assessment with each item ranked in term of 'risk or likelihood of future attempted suicide'. Based on their responses to these five items, pupils were assigned the single highest risk score at age 15 from the following; no risk factor = 0; recent (last month) thoughts of death or dying = 1; suicidal ideation (last year) = 2; recent (last month) suicidal ideation = 3; planned a suicide (last year) = 4; suicide attempt (ever) = 5. Additional file 1 provides details of the exact questions asked and risk score assigned to each item. The 2003 (age 19) nurse interview asked 'Have you ever tried to hurt yourself or harm yourself deliberately' and age at first act of self harm. Few (under 5%) reported self-harm before age 11, the majority (over 70%) of young people who self-harmed first did so during their secondary school years.

#### Prior mental health risk

At age 11, several indicators of early mental health problems or risk were recorded. Levels of depression and anxiety were assessed using the 6-item Kandel and Davies Depression Scale [37]. To assess previous psychiatric and major mental health problems, parents were asked about use of psychiatric services in relation to their child. Bullying and victimisation are strongly associated with mental health and, at 11, pupils were asked two questions about being bullied and teased (5-point scale, never to everyday) [38]. Pupils bullied or teased on a regular basis (weekly or more frequently) were categorised as being victimised.

#### Social and family background

In addition to gender, several other demographic and social background factors were recorded. Relative age has been linked with suicide [39] and the relative age of each pupil compared to cohort average (15 years, 5 months) at age 15 was calculated in months. Social class, obtained at age 11, was based on information about the occupation of the head of household, derived primarily from parents themselves, or, in the absence of a parental questionnaire, from reports by their children, which we have found to be reliable [40]. This was coded using the standard UK classification system [41] and categorized into social classes I-V, or missing. Although pupils reported parental social class at each study wave, social class was generally stable; accordingly, we include only baseline social class measures. At age 15 an area deprivation score, range 1 (least) to 7 (most deprived), was derived from pupils' postal codes using the 'Carstairs' index [42]. Religious affiliation was obtained from parents at baseline and categorized as Church of Scotland (Protestant - the majority religion and the established church in Scotland), Catholic (the second largest religion in Scotland), other (Muslim, Jewish, Methodist, Baptist, etc.), 'none, atheist/agnostic', or missing. At age 15, family structure was coded as 2-parent, 1-parent and other (reconstituted, other relative, foster parent, or other carer); low frequencies ruled out the creation of a separate 'reconstituted' (one 'birth' parent and new partner) category. Perception of the quality of parental-child relations was assessed using the 8-item Brief Parental Bonding Instrument (PBI-BC) [43], self-administered at age 15. The instrument is highly reliable (Cronbach's alpha > 0.7 for each subscale), demonstrates considerable validity [44] and produces scores on two scales representing perceived parental care, e.g. 'My parents help me as much as I need' and control, e.g. 'My parents treat me like a baby'; this mirrors the factor structure of the full scale. We used principal component (varimax) analysis of the 8 PBI-BC items to calculate factor scores. This allowed us to simultaneously derive standardized scores for interpretability, centre the scales and produce two uncorrelated measures; thereby reducing potential collinearity issues.

#### Perception of local neighbourhood

Pupils completed 11 questions relating to perceptions of their residential neighbourhood, derived from questions used in prior studies of environment influences used by the authors [45,46], or based on items used to evaluate school environment, broadly similar to Rutter's 'school ethos' items [3], but adapted for neighbourhoods. These were rated on either 3-point (good, average or bad), or 4-point scales (strongly agree to strongly disagree). See Additional file 2 for details.

Principal component (varimax) analysis of 11 items measuring perception of the local neighbourhood at age 15 produced three factors accounting for 57% of the variance. These were labelled *neighbourhood cohesion*, e.g. 'I feel part of this area'; *neighbourhood safety and incivilities*, e.g. 'I feel safe in this area' and *neighbourhood facilities*, e.g. rating of 'places for young people to meet'. See Additional file 2 for details.

#### School connectedness and perceptions of school environment

Pupils completed a series of questions related to aspects of school connectedness, including perceptions of the school environment, teacher-pupil relationships, pupil involvement, and school engagement [47]. With one exception, all measures were obtained from pupils at age 15. Briefly, these were largely derived from Rutter like 'school ethos' or 'school climate' items [3], full details of these items and the underlying factors are available elsewhere [14]. In relation to environment, pupils rated various physical (playground, library, gym/sports facilities, quiet places, etc.) and teacher-related (teaching, teacher control, teacher enthusiasm, etc.) aspects of their school as 'good', 'average' or 'bad'. Factor analysis produced a single dominant *school environment *factor combining both aspects. School involvement and engagement was assessed via seven items, using 4-point (strongly agree to strongly disagree) scales. Factor analysis produced two factors, the first loaded highly with items reflecting *school involvement *(like school, feel part of school, etc.); the second with items reflecting *school (dis)engagement *(don't like school, school a waste of time, etc.). The quality of *teacher/pupil relationships *was assessed by a single question, 'how many teachers would you say you get on well with?' with the response options, 'all most', 'about half', 'a few', 'only one', or 'none'. Finally, to adjust for the potential perceptual bias or general negative affectivity linked to primary school experiences, a measure of *prior school engagement *was computed from three items (like school, school a waste of time, skip school) which pupils completed at age 11, prior to starting secondary school. A scale for each construct was produced by summing items loading on each factor, which were then centered.

#### School context

In order to measure *school context*, we computed the mean scores for every school connectedness measure for each of the 43 schools, although one school was omitted due to missing data. These were averaged within school to give an overall *school ethos *score. This 'collective perception' of school ethos may provide a broader, more 'objective' assessment of school context then individual perceptions. More objective measures of school context were also included; *school roll*, extracted from publicly available education reports and statistics for the years 1998/1999, divided into quintiles and ranked from small to large; *school denomination*, (Catholic or non-denominational) and a *school rating *score (1-9), based on evaluations of features such as 'welcome', 'organization', 'pupil behaviour', etc., made by research nurses on completion of the survey in each school.

#### Statistical analysis

Variables representing school connectedness and school context were centred, positive scores indicating poorer ratings, (less involvement or engagement, and fewer teachers known). A binary dummy variable indicated a mismatch between individual religion and school denomination. Mismatched pupils included Catholic pupils attending a nondenominational (non-Catholic) school and non-Catholic pupils (Protestant, other religious groups and those with no religious beliefs) attending a Catholic school. Each of these groups describes pupils exposed to some form of mismatch between individual and institutional religious orientation. Due to low frequencies and conceptual similarity in risk, we combined both mismatched groups.

Analyses used multilevel logistic regression to determine associations between both individual and contextual variables and attempted suicide, suicide-risk at age 15 and self-harm at 19. Initial estimates were obtained by iterative generalized least squares estimation using the software package MLwiN 2.20 [48]. The final multivariate models were estimated by Markov chain Monte Carlo methods [49]. In order to estimate the impact of the covariates on attempted suicide, suicide-risk and self-harm we calculated the ICC of both the null and fully adjusted models. Odds ratios for both univariate and multivariate (mutually adjusted for background factors) and age 11 indicators of mental health are reported. We explored the cross-level interaction between individual religion and school denomination using a dummy variable, its significance tested both univariately and multivariately by adding the term to the final adjusted model. Finally, in order to maximize power we used the ranked risk or likelihood of future attempted suicide' score (for the sake of brevity henceforth termed 'suicidality score') to test for an interaction using normal multilevel regression.

## Results

### Descriptive statistics

Table 1 shows the prevalence rates for attempted suicide (ever) by age 15 (6.1%), suicide-risk at age 15 (9.4%) and self-harm (ever) by age 19 (6.8%), as well as frequencies of categorical covariates. Table 2 reports the descriptive statistics for the continuous covariates. Most continuous covariates are factor scores, or have been centred. Descriptive statistics for deprivation and prior depression are reported uncentred, because they are established interpretable scales. The impact of missing data on centred covariates resulted in only trivial departures from zero and re-centering these made no substantive difference to results.

**Table 1:** Frequencies for categorical predictors at individual and school-level

Categorical variables	*N*	%
**Outcome variables (*n *= 1698)**		

**Any Attempted Suicide (age 15)**		
No attempt	1595	93.9
Attempt	103	6.1
**Highest Suicide risk (age 15)**		
No risk	1539	90.6
Risk (attempt or ideation)	159	9.4
**Any Deliberate Self Harm (DSH) (age 19) [737 missing]**		
No DSH	915	93.2
DSH	67	6.8

**Individual level (*n *= 1698)**		

**Used Psychological service (age 11)**		
No	1470	86.6
Yes	49	2.9
Miss	179	10.5
**Bullied or tease (age 11)**		
Never	904	53.2
Less often	557	32.8
Weekly	237	14.0
**Gender**		
Female	856	50.4
Male	842	49.6
**Social Class (age 11)**		
I	103	6.1
II	432	25.4
IIIn	223	13.1
IIIm	530	31.2
IV	241	14.2
V	90	5.3
Missing	79	4.7
**Religion (age 11)**		
Protestant	642	37.8
Catholic	529	31.2
Other	115	6.8
None	229	13.5
Missing	183	10.8
**Family (age 15)**		
2-parent	1247	73.4
1-parent	267	15.7
Other (reconstituted or other family)	184	10.8

**School-level (*n *= 42) [1 missing]**		

**School roll**		
Q1	9	21.4
Q2	9	21.4
Q3	9	21.4
Q4	7	16.7
Q5	8	19.0
**Denomination**		
Non-denomination school	26	61.9
Denomination school	16	38.1

**Cross-level interaction (*n *= 1698)**		

**Religion & school denomination**		
Matching religion & denomination	1643	96.8
Mismatching religion & denomination	55	3.2

**Table 2:** Descriptive statistics for continuous predictors at individual and school-level

Continuous variables (age 15)	Mean	SD
**Individual level (*n *= 1698)**		

Age 11-Depression	15.54	3.51
Relative age (older than peers)	0.17	3.65
Deprivation (1-7 score)	4.11	1.94
Parental control (centred)	-0.01	1.56
Parental care (centred)	0.01	1.56

**School perceptions (*n *= 1698)**		

Age 11-engagement	-0.05	0.84
Environment (poorer)	-0.09	4.33
Involvement (poorer)	-0.09	1.98
(Dis)engagement (poorer)	-0.10	1.92
T/P relations (poorer)	-0.03	1.02

**Neighbourhood perceptions (*n *= 1698)**		

Neighbourhood cohesion (poorer)	0.00	0.99
Neighbourhood safety/civility (poorer)	-0.01	1.00
Neighbourhood facilities (poorer)	-0.02	1.01

**School level (*n *= 42)**		

Overall ethos (poorer)	0.14	0.84
School rating (poorer)	-0.01	0.47

### Univariate results

Tables 3, 4 and 5 show relationships between each covariate (grouped by type) and each outcome as univariate odds ratios. As expected, age 11 depression and victimisation were significantly associated with all outcomes, but psychiatric service use at or before age 11 was significantly associated with only age 15 suicide-risk. Perception of parental care and control were associated with respectively a lower and a higher likelihood of suicidality and self-harm. With the exception of our focus on religion, social background covariates were included as control variables and accordingly we do not comment further on their interpretation. In univariate analyses, religion was unrelated to suicidality or self-harm. With the exception of age 11 school engagement, measures of (poor) school connectedness were associated with increased odds in all outcomes. The majority of theses association were significant, with the remainder marginally significant. Unfavourable perceptions of neighbourhood cohesion and safety/incivilities, but not perceptions of neighbourhood facilities, were also associated with each suicide, but not self-harm outcome. Of the school level contextual covariates, the only significant univariate association was that between (poor) overall school ethos and self-harm at age 19 (OR 1.45, *p *= 0.029).

**Table 3:** School and individual effects on attempted suicide at age 15, unadjusted and fully adjusted models

Predictor	Attempted suicide Unadjusted OR	*p*	Attempted suicide Adjusted OR	*p*
**Prior mental health risk**				

**Age 11-Depression**	**1.14 (1.09-1.21)**	**< 0.001**	**1.10 (1.04-1.17)**	**0.001**
**Age 11-Psych service (no)**				
Yes	1.88 (0.72-4.85)	0.195	1.69 (0.59-4.87)	0.329
Missing	1.40 (0.78-2.52)	0.262	2.05 (0.33-12.88)	0.444
**Age 11-Victimised (never)**				
Less often	1.54 (0.98-2.43)	0.062	1.16 (0.71-1.90)	0.550
Weekly	**2.37 (1.40-4.01)**	**0.001**	1.59 (0.87-2.91)	0.129

**Social background**				

**Gender (male)**				
Female	**3.05 (1.94-4.78)**	**< 0.001**	**3.76 (2.27-6.23)**	**< 0.001**
**Relative age (older than peers)**	1.05 (0.99-1.11)	0.077	1.05 (0.99-1.12)	0.099
**Social Class (I)**				
II	0.70 (0.29-1.70)	0.430	0.56 (0.21-1.46)	0.233
IIIn	0.92 (0.36-2.35)	0.859	0.58 (0.20-1.68)	0.316
IIIm	0.91 (0.39-2.12)	0.828	0.59 (0.22-1.56)	0.288
IV	0.98 (0.39-2.45)	0.957	0.61 (0.21-1.75)	0.354
V	1.16 (0.39-3.43)	0.793	0.53 (0.15-1.85)	0.319
Missing	0.93 (0.28-3.04)	0.900	0.34 (0.08-1.37)	0.128
**Deprivation**	1.08 (0.97-1.20)	0.145	1.04 (0.90-1.20)	0.558
**Religion (Protestant)**				
Catholic	0.82 (0.50-1.34)	0.430	**0.39 (0.16-0.96)**	**0.041**
Other	0.67 (0.26-1.72)	0.402	0.65 (0.24-1.80)	0.406
None	1.03 (0.56-1.89)	0.931	0.91 (0.47-1.76)	0.785
Missing	1.21 (0.65-2.28)	0.546	0.46 (0.07-3.00)	0.418
**Family (2-parent)**				
Other	**2.64 (1.58-4.42)**	**< 0.001**	**1.96 (1.10-3.51)**	**0.023**
1-Parent	1.57 (0.93-2.66)	0.089	1.26 (0.70-2.27)	0.439
**Parental control**	**1.23 (1.09-1.38)**	**0.001**	1.03 (0.90-1.19)	0.641
**Parental care**	**0.76 (0.68-0.85)**	**< 0.001**	0.89 (0.77-1.03)	0.116

**School connectedness**				

**Age 11-engagement**	0.96 (0.76-1.23)	0.768	0.95 (0.72-1.25)	0.727
**Environment (poorer)**	**1.09 (1.04-1.14)**	**0.001**	0.02 (0.96-1.09)	0.567
**Involvement (poorer)**	**1.28 (1.17-1.41)**	**< 0.001**	1.14 (0.99-1.32)	0.077
**(Dis)engagement (poorer)**	**1.32 (1.19-1.45)**	**< 0.001**	1.15 (1.00-1.32)	0.055
**T/P relations (poorer)**	**1.38 (1.16-1.65)**	**< 0.001**	1.07 (0.85-1.36)	0.550

**Neighbourhood perception**				

**Neigh'd cohesion (poorer)**	**1.31 (1.08-1.58)**	**0.005**	1.06 (0.86-1.30)	0.579
**Neigh'd safety/civility (poorer)**	**1.31 (1.07-1.59)**	**0.008**	1.07 (0.84-1.36)	0.583
**Neigh'd facilities (poorer)**	1.12 (0.91-1.37)	0.285	0.94 (0.74-1.18)	0.582

**School level variables**				

**Overall (avg) ethos (poorer)**	1.25 (0.95-1.63)	0.105	0.89 (0.621.26)	0.499
**School roll (quintiles)**	1.07 (0.93-1.23)	0.330	1.09 (0.921.28)	0.312
**School rating (poorer)**	1.16 (0.76-1.78)	0.485	1.32 (0.822.14)	0.259
**Denomination**	1.00 (0.66-1.51)	1.000	**2.24 (1.014.99)**	**0.048**

**Cross-level interaction**				

**Mismatch between religion and school denomination**	**2.81 (1.30-6.10)**	**0.009**	-	-

Significant odds ratios are emboldened

**Table 4:** School and individual effects on suicide-risk at age 15, unadjusted and fully adjusted models

Predictor	Suicide risk Unadjusted OR	*p*	Suicide risk Adjusted OR	*p*
**Prior mental health risk**				

**Age 11-Depression**	**1.13 (1.08-1.18)**	**< 0.001**	**1.09 (1.04-1.15)**	**< 0.001**
**Age 11-Psych service (no)**				
Yes	**2.44 (1.15-5.15)**	**0.020**	2.28 (0.99-5.27)	0.054
Missing	**1.76 (1.11-2.80)**	**0.017**	2.63 (0.58-11.96)	0.210
**Age 11-Victimised (never)**				
Less often	1.28 (0.88-1.86)	0.193	1.03 (0.69-1.54)	0.902
Weekly	**2.01 (1.30-3.11)**	**0.002**	1.39 (0.84-2.30)	0.203

**Social background**				

**Gender (male)**				
Female	**2.66 (1.86-3.79)**	**< 0.001**	**3.33 (2.22-4.99)**	**< 0.001**
**Relative age (older than peers)**	1.03 (0.98-1.08)	0.199	1.03 (0.98-1.08)	0.261
**Social Class (I)**				
II	0.71 (0.36-1.38)	0.309	0.56 (0.27-1.18)	0.129
IIIn	0.61 (0.28-1.30)	0.202	**0.36 (0.15-0.87)**	**0.023**
IIIm	0.67 (0.34-1.30)	0.234	**0.41 (0.19-0.89)**	**0.025**
IV	0.62 (0.29-1.32)	0.212	**0.38 (0.16-0.92)**	**0.032**
V	1.17 (0.50-2.70)	0.721	0.53 (0.20-1.40)	0.197
Missing	0.77 (0.30-1.99)	0.588	**0.31 (0.10-0.96)**	**0.042**
**Deprivation**	1.03 (0.94-1.12)	0.551	0.98 (0.87-1.10)	0.710
**Religion (Protestant)**				
Catholic	1.47 (0.89-2.42)	0.135	0.51 (0.24-1.09)	0.084
Other	0.84 (0.54-1.29)	0.416	0.39 (0.15-1.03)	0.058
None	0.42 (0.16-1.07)	0.070	0.89 (0.51-1.55)	0.683
Missing	0.99 (0.59-1.65)	0.965	0.53 (0.11-2.43)	0.411
**Family (2-parent)**				
Other	**2.04 (1.30-3.21)**	**0.002**	1.54 (0.92-2.57)	0.101
1-Parent	1.43 (0.92-2.20)	0.109	1.13 (0.69-1.85)	0.633
**Parental control**	**1.28 (1.16-1.41)**	**< 0.001**	1.08 (0.96-1.21)	0.180
**Parental care**	**0.74 (0.67-0.81)**	**< 0.001**	**0.85 (0.76-0.96)**	**0.009**

**School connectedness**				

**Age 11-engagement**	1.01 (0.83-1.23)	0.922	1.00 (0.80-1.24)	0.983
**Environment (poorer)**	**1.08 (1.04-1.12)**	**< 0.001**	1.03 (0.98-1.08)	0.303
**Involvement (poorer)**	**1.21 (1.11-1.31)**	**< 0.001**	1.04 (0.92-1.17)	0.547
**(Dis)engagement (poorer)**	**1.30 (1.19-1.41)**	**< 0.001**	**1.18 (1.05-1.32)**	**0.006**
**T/P relations (poorer)**	**1.36 (1.17-1.57)**	**< 0.001**	1.09 (0.89-1.32)	0.419

**Neighbourhood perception**				

**Neigh'd cohesion (poorer)**	**1.23 (1.05-1.45)**	**0.009**	1.02 (0.85-1.21)	0.843
**Neigh'd safety/civility (poorer)**	**1.24 (1.05-1.46)**	**0.010**	1.05 (0.86-1.28)	0.641
**Neigh'd facilities (poorer)**	1.11 (0.94-1.32)	0.224	0.91 (0.76-1.10)	0.352

**School level variables**				

**Overall (avg) ethos (poorer)**	1.24 (1.00-1.55)	0.055	1.07 (0.80-1.42)	0.660
**School roll (quintiles)**	1.08 (0.96-1.22)	0.204	1.05 (0.92-1.21)	0.451
**School rating (poorer)**	1.39 (0.96-2.02)	0.081	**1.65 (1.10-2.47)**	**0.016**
**Denomination**	0.95 (0.65-1.38)	0.776	1.89 (0.95-3.78)	0.071

**Cross-level interaction**				

**Mismatch between religion and school denomination**	**2.09 (1.01-4.31)**	**0.047**	-	-

Significant odds ratios are emboldened

**Table 5:** School and individual effects on deliberate self-harm at age 19, unadjusted and fully adjusted models

Predictor	Self-harm Unadjusted OR	*p*	Self-harm Adjusted OR	*p*
**Prior mental health risk**				

**Age 11-Depression**	1.06 (0.99-1.14)	0.073	1.04 (0.96-1.13)	0.288
**Age 11-Psych service (no)**				
Yes	1.16 (0.27-5.05)	0.840	0.99 (0.20-4.85)	0.995
Missing	0.70 (0.25-1.99)	0.507	3.83 (0.26-55.93)	0.326
**Age 11-Victimised (never)**				
Less often	**1.92 (1.11-3.33)**	**0.020**	1.61 (0.88-2.95)	0.119
Weekly	1.80 (0.88-3.68)	0.107	1.34 (0.58-3.06)	0.493

**Social background**				

**Gender (male)**				
Female	1.56 (0.94-2.59)	0.082	1.54 (0.87-2.73)	0.141
**Relative age (older than peers)**	1.04 (0.97-1.11)	0.307	1.04 (0.96-1.12)	0.315
**Social Class (I)**				
II	1.61 (0.32-8.01)	0.561	0.51 (0.20-1.31)	0.160
IIIn	1.02 (0.23-4.61)	0.977	0.59 (0.19-1.80)	0.354
IIIm	1.47 (0.31-6.98)	0.628	**0.20 (0.06-0.64)**	**0.007**
IV	0.52 (0.11-2.54)	0.422	0.49 (0.15-1.64)	0.249
V	1.36 (0.28-6.53)	0.701	1.74 (0.49-6.22)	0.393
Missing	3.67 (0.74-18.26)	0.112	0.49 (0.07-3.62)	0.484
**Deprivation**	1.00 (0.88-1.14)	0.970	0.97 (0.80-1.18)	0.786
**Religion (Protestant)**				
Catholic	0.61 (0.32-1.15)	0.128	**0.07 (0.01-0.31)**	**0.001**
Other	1.57 (0.69-3.57)	0.288	1.18 (0.47-2.96)	0.724
None	1.08 (0.53-2.23)	0.825	1.00 (0.46-2.17)	0.994
Missing	0.44 (0.13-1.48)	0.186	0.03 (0.00-0.65)	0.026
**Family (2-parent)**				
Other	**2.50 (1.27-4.94)**	**0.008**	**2.72 (1.23-5.99)**	**0.013**
1-Parent	1.40 (0.68-2.85)	0.358	1.28 (0.57-2.88)	0.554
**Parental control**	**1.22 (1.05-1.42)**	**0.008**	1.12 (0.93-1.35)	0.217
**Parental care**	**0.78 (0.67-0.90)**	**0.001**	0.92 (0.76-1.12)	0.413

**School connectedness**				

**Age 11-engagement**	1.09 (0.81-1.47)	0.580	1.18 (0.84-1.65)	0.346
**Environment (poorer)**	1.05 (0.99-1.12)	0.074	1.03 (0.95-1.12)	0.469
**Involvement (poorer)**	**1.17 (1.03-1.32)**	**0.012**	1.01 (0.84-1.22)	0.892
**(Dis)engagement (poorer)**	**1.21 (1.07-1.38)**	**0.003**	1.16 (0.97-1.39)	0.113
**T/P relations (poorer)**	1.23 (0.98-1.56)	0.080	0.92 (0.67-1.28)	0.634

**Neighbourhood perception**				

**Neigh'd cohesion (poorer)**	1.25 (0.98-1.59)	0.077	1.06 (0.80-1.41)	0.680
**Neigh'd safety/civility (poorer)**	1.04 (0.82-1.34)	0.729	0.88 (0.65-1.19)	0.400
**Neigh'd facilities (poorer)**	1.06 (0.83-1.36)	0.632	1.05 (0.79-1.39)	0.741

**School level variables**				

**Overall (avg) ethos**	**1.45 (1.04-2.03)**	**0.029**	1.31 (0.85-2.01)	0.221
**School roll (quintiles)**	1.10 (0.92-1.31)	0.281	1.05 (0.84-1.30)	0.671
**School rating**	0.82 (0.47-1.44)	0.496	0.96 (0.50-1.82)	0.896
**Denomination**	0.81 (0.47-1.40)	0.457	**9.15 (2.17-38.53)**	**0.003**

**Cross-level interaction**				

**Mismatch between religion and school denomination**	**4.12 (1.32-12.89)**	**0.015**	-	-

Significant odds ratios are emboldened

### Multivariate results

Tables 3, 4 and 5 also show the multivariate associations between each covariate and each suicidality outcome resulting from fully adjusted (adjusted for all covariates) models. Depression at age 11 was associated with increased odds of attempted suicide and suicide-risk at age 15, but not self-harm at age 19. Use of psychiatric services at or before age 11 was associated with only age 15 suicide-risk (OR 2.28, *p *= 0.05), whereas victimisation was unrelated to any outcome. Compared to Church of Scotland (Protestant) pupils, Catholic pupils had lower odds of attempted suicide (OR 0.39, *p *= 0.04), suicide-risk (OR 0.51, *p *= 0.08) at age 15 and of self-harm (OR 0.07, *p *= 0.001) at 19. After adjusting for all the variables in the model, perceptions of the local neighbourhood were not related to any of the outcomes. For all three outcomes, school (dis)engagement was associated with a 15-18% increase in odds for every SD above the average; significantly for suicide-risk (*p *= 0.006) and near significantly for attempted suicide (*p *= 0.055) at age 15. Poor school involvement was also associated with a similar, but non-significant (*p *= 0.077), increase in the odds of attempted suicide by age 15. A poor school rating was associated with a significant increase in the odds of suicide-risk by age 15 (OR 1.65, *p *= 0.016). In contrast to the findings for individual religion, when compared to pupils attending a non-denominational school, those attending a denominational (Catholic) school had a substantial increase in the odds of attempting suicide (OR 2.24, *p *= 0.048), suicide-risk by age 15 (OR 1.89, *p *= 0.071), and self-harm by age 19 (OR 9.15, *p *= 0.003).

### Cross-level interactions

The apparent paradoxical result that individual Catholic religion is a protective, but Catholic school attendance a risk factor, can be explained by examining their cross-level interaction. Table 6 shows the cross-tabulation between these two variables. Where there is a 'mismatch' between religion and denomination there is approximately a two to fourfold increase in the rates of attempted suicide, suicide-risk and self-harm and this is reflected in the univariate odds ratios (Tables 3, 4 and 5). Adding this 'religious mismatch' interaction to the adjusted model removed both the protective effect of individual (i.e. catholic advantage) religion and the increased risk associated with denominational school, although in the fully adjusted model the interaction was not significant. To explore if this lack of significance for the interaction in the adjusted model was attributable to low statistical power, the analysis was repeated using standard multilevel regression with the 'pseudo' continuous suicidality score as the outcome; in that multivariate analysis the interaction was significant (*p *= 0.03, 1-tail). Excluding pupils from minority religion (Muslim, Jewish, Methodist, Baptist, etc.) made no substantive difference to results; therefore it is unlikely that our results are attributable to the influence of minority religions.

**Table 6:** Relationship between individual religion, school denomination and attempted suicide, suicide-risk and self-harm

Outcome	Religion & school denomination		
	
	Match% (*N*)	Mismatch% (*N*)	*p*
**Attempted suicide (age 15)**	5.8 (95)	14.5 (8)	0.016
**Suicide risk (age 15)**	9.1 (150)	16.4 (9)	0.070
**Self-harm (age 19)**	6.5 (63)	22.2 (4)	0.029

### School-level variance

In line with previous studies of school and 'wellbeing' the estimated percent of variance attributable to school in the null model was low - less than 1% for attempted suicide, 1.3% for suicide-risk, and 1.6% for self-harm and this altered little in the fully adjusted models.

## Discussion and Conclusions

Returning to the first of our three aims, after adjusting for social background we found several significant associations between suicidality or self-harm and school connectedness (school engagement) and school-level (school denomination) variables. However, many associations were non-significant, although of broadly of similar effect size. In relation to our second aim, we found evidence for a cross-level interaction between individual religion and school denomination compatible with Durkheim's theory of suicide. In relation to our final aim and in line with previous studies of 'psychological wellbeing', we found that very little of the variation in attempted suicide, suicide-risk or self-harm is attributable to the school level. If replicated, these findings have important implications for suicidology, school-based public health policy and interventions aimed at reducing youth suicide and self-harm, although we explore several alternative explanations for our findings.

Several covariates behave differently in the adjusted models, only becoming significant in multivariate analyses, most notably social class and religion/school denomination and this requires explanation. Unadjusted, social class seems largely unrelated to suicide-risk and self-injury, but the two are occasionally associated in the adjusted models. This is likely attributable, at least in part, to statistical artefact and the use of a relatively small reference group. Children from privileged backgrounds - social class I (doctors, lawyers, academics, etc.) appear to be at increased risk. While traditionally a low-risk group, children of high social class parents, especially females, may feel additional academic pressure during adolescence because of higher parental- and self-expectations [50]. In the unadjusted models, religious upbringing and the denomination of the school attended are unrelated to suicidality, but this dramatically changes in the multivariate models, producing an apparent paradox; being Catholic generally reduces, while attending a Catholic school increases the risk of suicide. We can explain this contradiction by an intriguing interaction; Catholic pupils attending Catholic schools report normal or slightly lower, but non-Catholic pupil attending denominational (Catholic) schools report substantially higher levels of suicidality, not only while at school but also after school leaving, and vice versa for Catholic pupils attending non-denominational schools. It appears that pupils whose religion and school denomination are at odds are at greater risk.

Although social background is a major influence, our results suggest that the school environment matters for mental health; more specifically, pupils with low levels of school engagement and involvement are more likely to attempt or seriously think about taking their own life or deliberately harm themselves. Irrespective of any theoretical perspective and accepting the limitation of our measures, our most striking finding is that pupils who go to a school with a religious perspective incongruent with their own are approximately twice as likely to or attempt or think about suicide and four times more likely to self-harm.

### Durkheim's theory and alterative explanations

Durkheim's theory of suicide proposes that egoistic suicides are increased when individual connections to society are weak and anomic suicides are increased by 'mismatch' between personal and societal norms and expectations. In relation to suicide-risk and self-harm, the 'protective' effect of school connectedness and the increased risk to pupils of a different religious orientation from their school or the religion of the majority of pupils are two findings highly compatible with egoistic and anomic suicide perspectives respectively. However, there are alterative explanations for increased suicidality among 'mismatch' pupils. Parents sending their children to a school whose 'ethos' significantly differs from their religion may do so for reasons unrelated to religious belief; for example parents focused on their child's scholastic success rather than wellbeing may choose a school based on school performance over other considerations. The inclusion of social background and parental care in our analyses may have reduced the impact of such unmeasured factors. We also cannot exclude the influence of pre-existing mental health problems or risk factors, although we included covariates that should have reduced the impact of such confounds. Ethnicity may play a role, since certain minority groups show high rates of self-harm [51] and some (non-Catholic) minority parents prefer to have their child(ren) educated within a Catholic school. We explored this in further (unreported) analysis and found ethnicity did not explain the high rates of suicidal and self-harm behaviours in the 'mismatched' group. The West of Scotland has a history of sectarianism, and therefore the targeting and victimisation of pupils of a different religion [23] is another possible explanation for increased mental health issues among 'mismatched' pupils, although increased risk of victimisation can be interpreted as further evidence of the negative consequence of not sharing institutional norms.

Regarding the ecological transactional model of suicide, we replicated the relatively weak protective effects of the exosystem (neighbourhood) and stronger effects of the microsystem (school connectedness and parental attachment) on suicidal behaviour reported by Kidd et al. [27]. Further, the interaction between religion and school denomination is in-line with the importance that the ecological transactional model places on interactions between ecological levels. Our findings are also compatible with Joiner's Interpersonal Theory of Suicide since it is plausible that, in combination with other risk factors, pupils who feel uncared for by parents, or who perceive greater exclusion by peers because of religious or other differences are likely to experience elevated levels of thwarted belongingness and perceived burdensomeness. Importantly because Joiner's theory is a cognitive behavioural one, individual *self-perceptions *of isolation should be more important as risk factors than objective ecological measures of *actual *levels of exclusion and our findings are compatible with this more cognitive perspective.

Several standard methodological caveats apply to our findings. It is always possible to omit important variables by accident or through limitations of the dataset. At the other extreme, collinearity may be an issue. This may be particularly relevant to the school connectedness and school ethos measures because of the ephemeral nature and conceptual overlap of these constructs. To partially address this, we used measures derived through exploratory factor analysis, which may help to minimize, although not completely eliminate major collinearity problems [14]. Our study design is a practical one containing elements of both cross-sectional and longitudinal designs and thus imperfect; it does not fully track all suicide-risk outcomes and covariates at every time point - an ideal study would do so, allowing analysis of complex trajectories and their interaction with covariates.

Despite the low prevalence of our outcomes, our study has adequate power to detect even small main effects, although power to detect interactions is modest. Nevertheless, the consistency of findings across all three suicidality outcomes and the significant interaction found between religion and denomination using our suicidality measure suggests this is a reasonably robust effect, at least in this cohort. We plan to replicate this analysis using more general measures of mental health, which should provide additional power to explore such interactions [23].

#### Policy implications & conclusions

Currently, there is growing social and political pressure to allow both denominational (alternatively called faith schools), and independent (private or fee paying) schools greater freedom in shaping their own 'ethos and values' and our results may have potentially serious implications for school policy. Two extreme policies are sometimes proposed; the first is a return to religious segregation within schools; this may reduce the levels of anomie, but is problematic in terms of promoting diversity and tolerance agendas. The second is the removal of all religious distinctions within school; while this may also lower levels of anomie, many parents and religious leaders are strongly committed to retaining denominational (faith) schools, and any steps to remove religious organisations from the educational system are likely to be characterised as an infringement of civil freedom and parental choice. A 'middle way' would be to continue with current practice, but consider 'religiously mismatched' young people at increased risk for suicidality and, where possible, schools could implement policies to minimise the mismatch between the values of the pupils and school. In practice, this is difficult to implement since pupils may 'home in' on such distinctions and such policies risk further emphasising existing differences. Policies which aim to strengthen concepts such as school connectedness as a prophylactic are uncontroversial and this study offers additional supporting evidence for their efficacy [18].

Although our results make intuitive sense and are highly compatible with both Durkheim's anomic and Joiner's ITS account of suicide and are broadly in-line the E-TM framework, we are appropriately cautious in our conclusion. While we speculate about policy implications, this is a single study from a location with a history of sectarianism and how generalizable these findings are beyond the 'West of Scotland' context has yet to be established.

## Competing interests

All authors are supported financially by the Medical Research Council of Great Britain. RY and AE as part of the Neighbourhoods and Health (WBS U.1300.00.009) and HS as part of the Gender and Health program (WBS U.1300.00.004). The authors declare no competing interests.

## Authors' contributions

RY wrote the manuscript, conceived the theoretical approach taken in the paper, participated in design of the final phase of the 11-16/16+ Study, and performed the statistical analysis. HS co-conceived the original 11-16/16+ Study, participated in its design and coordination and contributed to drafting the manuscript. AE contributed to the neighbourhoods section of the manuscript. All authors read and approved the final manuscript.

## Pre-publication history

The pre-publication history for this paper can be accessed here:

http://www.biomedcentral.com/1471-2458/11/874/prepub

## Supplementary Material

Additional file 1 **Table S1. Suicide risk score Relationship between individual items and suicidality score**. Click here for file

Additional file 2 **Table S2: Factor analysis of 11 'perceptions of neighborhood' items**. Click here for file
