# Supplementary material for: Do schools differ in suicide risk? the influence of school and neighbourhood on attempted suicide, suicidal ideation and self-harm among secondary school pupils
Source: BMC Public Health. 2011 Nov 17;11:874. doi: 10.1186/1471-2458-11-874 (PMC3280202; doi:10.1186/1471-2458-11-874)
Supplement: Additional file 1 — Table S1. Suicide risk score Relationship between individual items and suicidality score. [file 1471-2458-11-874-S1.DOC]

**Table S1. Suicide risk score Relationship between individual items and suicidality** score

| **Item** | **Suicidality Score** |
| --- | --- |
| **No suicide risk factor** | **0** |
| **Recent (last month) thoughts of death or dying**  Item: *‘In the last four weeks, have you often thought about death or about people who have died or about being dead yourself?’* | **1** |
| **Suicidal ideation (last year)**  Item: *‘In the last year, was there a time when you thought seriously about killing yourself?’* | **2** |
| **Recent (last month) suicidal ideation**  Item: *‘What about the last four weeks, have you thought seriously about killing yourself?’* | **3** |
| **Planned a suicide (within last year)**  Item: *‘In the last year, did you have a plan for exactly how you would kill yourself?’* | **4** |
| **Suicide attempt (ever)**  Item: *‘Have you ever, in your whole life, tried to kill yourself or make a suicide attempt?’* | **5** |
