# Supplementary material for: Do schools differ in suicide risk? the influence of school and neighbourhood on attempted suicide, suicidal ideation and self-harm among secondary school pupils
Source: BMC Public Health. 2011 Nov 17;11:874. doi: 10.1186/1471-2458-11-874 (PMC3280202; doi:10.1186/1471-2458-11-874)
Supplement: Additional file 2 — Table S2: Factor analysis of 11 'perceptions of neighborhood' items. [file 1471-2458-11-874-S2.DOC]

**Table S2: Factor analysis of 11 ‘perceptions of neighborhood’ items**

|  |  | **Label** |  |
| --- | --- | --- | --- |
| **Questionnaire item** | **Cohesion** | **Safety & incivilities** | **Facilities** |
| I feel part of this area** | .77 |  |  |
| I want to leave this area** | -.72 |  |  |
| I like the people in this area** | .72 |  |  |
| I like this area** | .70 |  |  |
| Rate: *the safety of the area* * |  | .81 |  |
| Rate: *the overall tidiness of the area* * |  | .81 |  |
| I feel safe in this area** |  | .67 |  |
| Other people think this is a good area ** |  | .63 |  |
| Rate: *sports facilities* * |  |  | .78 |
| Rate: *places for young people to meet* * |  |  | .69 |
| Rate: *public transport* * |  |  | .65 |

Principal components with varimax rotation.

Loadings below 0.5 suppressed and leading zeros omitted.

* 3-point scale (good, average or bad); ** 4-point scale (strongly agree to strongly disagree).
